# Thermal, Thermomechanical, and Fire Behaviour of Casein-Modified Unsaturated Polyester Resin Biocomposites

**DOI:** 10.3390/polym18202552

**Published:** 2026-10-16

**Authors:** Przemysław Pączkowski, Karolina Głogowska, Bronisław Samujło, Barbara Gawdzik

**Affiliations:** 1 Department of Polymer Chemistry, Institute of Chemical Sciences, Faculty of Chemistry, Maria Curie-Sklodowska University, Gliniana 33, 20-614 Lublin, Poland; barbara.gawdzik@umcs.pl; 2 Department of Technology and Polymer Processing, Faculty of Mechanical Engineering, Lublin University of Technology, Nadbystrzycka 36, 20-618 Lublin, Poland; k.glogowska@pollub.pl (K.G.); b.samujlo@pollub.pl (B.S.)

**Keywords:** unsaturated polyester resin, casein, environmentally friendly composites, bio-based flame retardant, thermal properties, cone calorimetry, degradation studies

## Abstract

The increasing demand for sustainable engineering thermosets, such as unsaturated polyester resins (UPR), together with stricter fire safety requirements, promotes the development of environmentally friendly polymer composites. In this study, UPR synthesized from recycled post-consumer poly(ethylene terephthalate) was modified with casein particles to investigate their effects on thermal, thermomechanical, and fire-related properties. The obtained biocomposites were characterized by thermal analysis, thermomechanical testing, and flammability evaluation. Casein incorporation affected the degradation behaviour of UPR and promoted residue formation at elevated temperatures. Cone calorimetry demonstrated a reduction in peak heat release rate from 600 to 407 kW·m^−2^ and total heat release from 86 to 65 MJ·m^−2^ for the composite containing 15 wt.% casein. The maximum average rate of heat emission decreased from 345 to 255 kW·m^−2^, while combustion residue increased from 0.1 to 3.5%, confirming enhanced char formation. Fire performance also improved, with the Fire Potential Index increasing from 0.053 to 0.071 m^2^·s·kW^−1^ and the Fire Retardancy Index reaching 1.77 for UPR+C15. The heat deflection temperature increased from 56.9 to 71.3 °C, whereas the Vicat softening temperature decreased slightly. Overall, casein is a promising bio-based additive for tailoring the properties of sustainable UPR composites.

## 1. Introduction

Due to their numerous advantages, polymeric materials and polymer-based composites are widely used in a broad range of industrial applications. However, the majority of polymers are inherently flammable, which limits their use in applications where enhanced fire safety is required. To meet relevant fire safety standards, flame retardants are commonly incorporated into polymeric materials that do not inherently provide the required level of fire resistance [1,2].

Traditionally, the ignition and flame resistance of resin products has been improved by adding halogenated flame retardants such as tetrabromophthalic anhydride and antimony trioxide. However, increasing environmental and health concerns have led to a growing interest in halogen-free alternatives. These additives are now replaced by halogen-free compounds such as phosphorus compounds e.g., tris(2-chloroethyl) phosphates, tris(2,3-dibromo propyl)-tricresol phosphates, metal oxides and hydroxides, zinc and tin compounds, nitrogen compounds, boron compounds, and swelling systems that create coatings on the burning plastic surface to prevent heat penetration and gas diffusion. The synergistic flame retardant effects of certain flame retardants, such as nitrogen and phosphorus, antimony oxides with halogen compounds, and bromine and phosphorus, are also been widely investigated [3,4,5]. Kicko-Walczak and Rymarz reported the use of various halogen-free flame retardants in composite materials based on unsaturated polyester and epoxy resins [6,7].

Despite their effectiveness, the incorporation of flame retardants may adversely affect the mechanical and processing properties of polymeric materials. Flame retardants reduce fire hazard and ensure the required fire safety level. They have been applied for decades and offer benefits and shortcomings. The efficacy of flame retardants in decreasing the flammability of polymeric materials depends on a number of factors, such as the type of a polymeric material, its application and exposure conditions, as well as their compatibility with a given material and its processability. Some flame retardants may release toxic gases during combustion, while others may also cause the accelerated breakdown of materials [8]. Certain additives are hygroscopic and may promote hydrolysis, whereas others need to incorporated at relatively high concentrations to achieve the desired flame-retardant effect. Consequently, the addition of flame retardants may negatively affect the mechanical properties of polymer matrix and the interfacial adhesion between the components of composite materials. Therefore, current research increasingly focuses on the development of flame-retardant systems that combine effective fire protection with improved environmental and health safety while maintaining the functional properties of the polymeric material.

Bio-based materials are of particular interest as potential alternatives to conventional flame-retardant additives. Casein appears to be a promising candidate due to its bio-based origin and potential to reduce the flammability of polymeric materials [9,10,11,12]. However, despite its potential as a halogen-free flame-retardant additive, the application of casein in polyester resin systems, particularly unsaturated polyester resins (UPR), has not been sufficiently investigated. Considering the widespread use of UPRs, the development of effective and environmentally friendly approaches to improve their flame-retardant properties remains an important research challenge.

The aim of the present study was to investigate the potential of fine casein powder as a bio-based flame-retardant additive in unsaturated polyester resin-based biocomposite materials. The study focused on the effect of casein content on the fire behaviour of the resulting composites. Casein was selected as the flame-retardant additive due to its bio-based origin, low cost, availability, and potential to provide a more environmentally friendly alternative to conventional halogen-containing flame retardants.

## 2. Materials and Methods

### 2.1. Materials

An unsaturated polyester resin (UPR) based on post-consumer recycled poly(ethylene terephthalate) (14PB-06) was supplied by LERG (Pustków, Poland). Methyl ethyl ketone peroxide (MEKP, Luperox DHD-9) used as an initiator was obtained from Sigma-Aldrich (St. Louis, MO, USA). A 4% solution of an environmentally friendly polymeric cobalt accelerator was synthesized at Department of Polymer Chemistry of Maria Curie-Skłodowska University (Lublin, Poland) [13].

Casein (fine powder, 90 mesh; max. 83.5%, protein, 12.0% water, 2.5% fat, 2.5% ash, and 0.7% free acid) was supplied by Kremer Pigmente GmbH & Co. KG (Aichstetten, Germany).

### 2.2. Sample Preparation

The resin was cured using a system consisting of MEKP (1.1 wt.%) and polymeric cobalt (0.25 wt.%) was used. Identical quantities of initiator and the accelerator were used to prepare casein-resin biocomposites. The materials were prepared by mixing UPR with different filler contents up to 15% (*w*/*w*), denoted as UPR+C5, UPR+C10, and UPR+C15, corresponding to 5%, 10%, and 15% (*w*/*w*) filler content, respectively.

To ensure good homogeneity, all ingredients were mixed and degassed in a vacuum chamber from VacuumChambers.eu (Białystok, Poland) at a pressure of 1 bar and room temperature (~23 °C). After that, they were poured into cuboid moulds consisting of two glass panes coated with a thin anti-adhesive layer and having a 4 mm thick Teflon spacer between them. They were cured at room temperature for 24 h and then post-cured at 80 °C for 10 h. Obtained cuboid-shaped samples were cut to the required dimensions on a CNC milling machine type MFG 8037P manufactured by Ergwind (Gdańsk, Poland).

### 2.3. Morphological Characterization

The morphology of UPR-casein composite samples was characterized using a FEI Quanta 3D FEG high-resolution scanning electron microscope (Hillsboro, OR, USA). The microscope was operated in high-pressure mode at accelerating voltages of 5 and 20 kV, depending on the ample and imaging conditions. The images were acquired using an Everhart–Thornley detector (ETD). Prior to examination, the samples were sputter-coated with a thin palladium/gold (Pd/Au) layer using a Quorum Technologies Polaron SC7640/CA7625 sputter coater to prevent electrostatic charging during SEM analysis (Quorum Technologies Ltd, Bicester, UK).

### 2.4. Thermal and Thermomechanical Properties

The thermal properties of the UPR/casein samples were determined by thermogravimetry, differential scanning calorimetry, as well as Vicat softening temperature (VST) and heat deflection temperature (HDT) evaluation.

The thermal stability of the UPR/casein samples was assessed via thermogravimetry (TG) and differential scanning calorimetry (DSC). TG/DTG/DSC data were acquired using the Netzsch STA 449F1 Jupiter Simultaneous Thermal Analyzer (Selb, Germany), in compliance with the procedure specified in the EN ISO 11358-1 standard [14]. The sample mass was about 10 mg. The heating range was 30–1000 °C at a heating rate of 10 °C min^−1^ in an oxidative atmosphere (synthetic air).

The Vicat softening temperature (VST) of the samples was evaluated with the Ceast HV3 apparatus from Instron (Turin, Italy), in compliance with EN ISO 306:2013 [15] by method B120, using a force of 50 N and a heating rate of 120 °C h^−1^. The heat deflection temperature (HDT) of the test samples was determined with the use of the same apparatus, following the procedure delineated in EN ISO 75-2:2013 [16]. This was done via method C, using a flexural stress of 8 MPa and a heating rate of 120 °C h^−1^, with the deflection value set to 0.36 mm.

### 2.5. Cone Calorimetry Studies

The samples were studied for their fire properties using a cone calorimeter (Fire Testing Technology Ltd., East Grinstead, UK) in accordance with EN ISO 5660-1:2015 [17]. Specimens with dimensions of 100 × 100 × 4 (mm^3^) were tested at a heat flux of 35 kW m^−2^. The following data were obtained: time to ignition (TTI), peak heat release rate (pHRR), total heat release (THR), total smoke production (TSP), maximum average heat release rate (MARHE), fire potential index (FPI), fire retardancy index (FRI) and residue. The experiments were carried out for 1200 s. Three specimens from each sample were tested and the average results are reported.

### 2.6. Flammability Tests

Flammability was conducted in accordance with EN ISO 60695-11-10. Part 11-10 [18] using a universal flammability tester (Lublin University of Technology, Lublin, Poland). The specimens were tested in both horizontal and vertical orientation with a 50 W propane burner as the ignition source. Changes in the thermal field during combustion were monitored using a FLIR A70 thermal imaging camera (Wilsonville, OR, USA). Thermal images were recorded from a distance of 1 m immediately after removal of the ignition source at time intervals from 10 to 90 s. The acquired thermal images (thermograms) were analyzed using FLIR Research Studio Player software (v.1.7.)

For the horizontal burning test (method A), the specimen was clamped horizontally. The burner was inclined at an angle of 45° to the specimen, and the flame was applied to the lower free end of the specimen to achieve an impingement length of approximately 6 mm. The ignition flame was maintained for 30 ± 1 s, after which the burner was removed. Once the flame front reached the start mark of the measuring section, the burning time required for the flame to travel the 50 mm measuring length was recorded. For linear burning rate *v* (mm min^−1^), was calculated according to Equation (1):

(1) $$v=\frac{60\cdot L}{t},$$

where *L* is the damaged (burned) length of the specimen (mm) and *t* is the burning time (s). For the specimen dimensions used in this study, the measuring length was 50 mm.

For the vertical burning test (method B), the specimens were mounted vertically and tested in accordance with the procedure specified in EN ISO 60695-11-10 [18].

### 2.7. Data Analysis

The experimental data were analyzed using appropriate statistical methods. For the methods requiring replicate measurements, the results are expressed as mean values ± standard deviation (SD). Statistical significance was assessed using one-way analysis of variance (ANOVA), followed by Tukey’s honestly significant difference (HSD) multiple-comparison test. Differences were considered statistically significant at *p* < 0.05.

## 3. Results and Discussion

Casein is the most important component of protein in bovine milk, accounting for approximately 80% of the total milk protein content [19]. It is a naturally occurring protein-based biopolymer typically obtained as a white to yellowish slightly hygroscopic powder. Due to its renewable origin, biodegradability, and functional chemical structure, casein has attracted increasing attention as a sustainable additive for polymer-based materials. Casein is classified as a phosphoprotein because its structure contains approximately 0.7–0.9 wt.% phosphorus [20].

Casein is not a single uniform compound but a complex mixture of αs_1_-, αs_2_-, β-, and κ-casein fractions, present at 40, 10, 35, and 15 wt.%, respectively [21]. Due to hydrophobic, hydrogen and electrostatic bonding, casein molecules are linked via the protein–protein bonds. Their linkage is also affected by the presence of calcium and phosphate (6–8 wt.%) in the micelle structure [21]. About 3% of casein is present in a colloidal milky opalescent solution. A small percentage of the population is allergic to casein, a condition that is known as milk protein intolerance [22].

The incorporation of casein into the UPR matrix was investigated to evaluate its influence on the morphology, thermal behaviour, thermomechanical properties, and fire performance of the obtained composites. Due to its protein-based structure containing phosphorus-containing groups and its ability to form thermally stable carbonaceous residues during degradation, casein may contribute to changes in the decomposition mechanism and combustion behaviour of polymer composites.

The mechanical and surface properties of the UPR/casein composites, including flexural and impact strength, gloss, and water contact angle, have been previously investigated and reported by the authors [23]. Therefore, these aspects are not repeated in the present study, which focuses primarily on the thermal and fire performance of the developed sustainable polyester composites.

### 3.1. Morphology of Casein-Modified UPR Biocomposites

**Figure 1:**
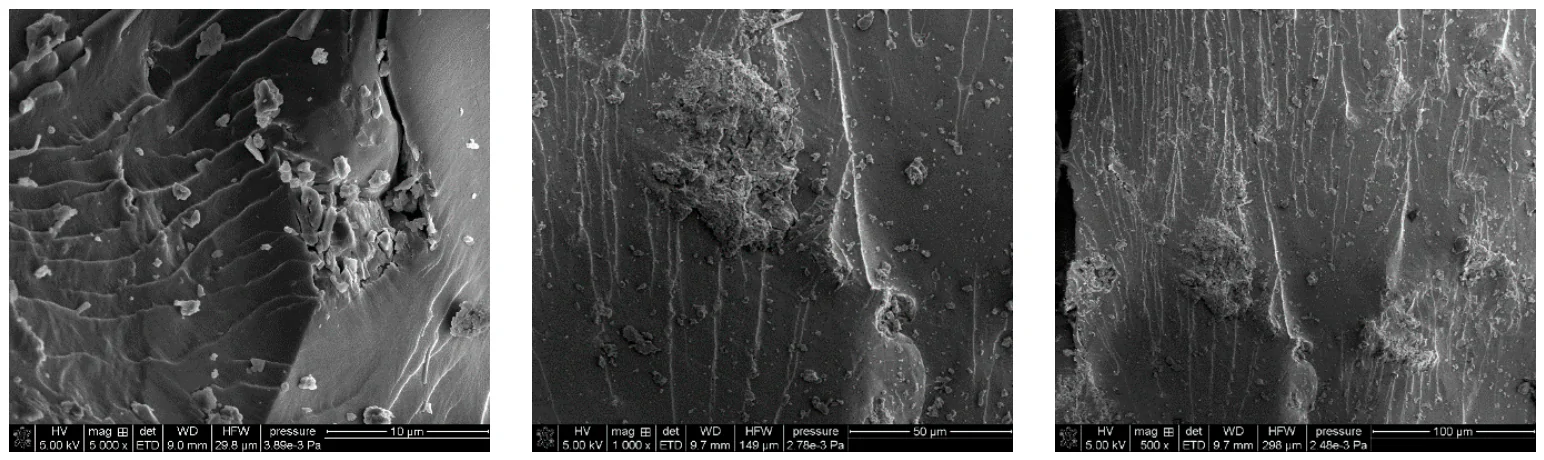
SEM micrographs of the UPR+C15 composite showing the morphology and distribution of casein particles within the unsaturated polyester matrix at different magnifications (5000×, 1000×, 500×).

SEM analysis was performed to evaluate the morphology and distribution of casein particles within the unsaturated polyester resin (UPR) matrix. Representative micrographs of the UPR+C15 composite obtained at different magnifications are presented in Figure 1.

The SEM images confirm the successful incorporation of casein into the UPR matrix and reveal the heterogeneous morphology of the obtained composite. At lower magnification, the continuous polymer phase containing dispersed casein-rich regions can be observed, indicating that the filler was distributed throughout the composite structure. The absence of extensive void formation or large regions of interfacial separation suggests relatively good compatibility between the casein particles and the polyester matrix.

At higher magnifications, individual casein-rich domains and locally aggregated regions can be identified. The formation of such clusters is typical for polymer composites containing bio-based particulate fillers and may result from the strong intermolecular interactions between protein molecules. Nevertheless, the observed morphology indicates that casein particles remain embedded within the polymer network and maintain contact with the surrounding resin phase.

The morphology of the composite is an important factor affecting its thermal and fire behaviour. The dispersed casein phase may contribute to the formation of thermally stable structures during decomposition, acting as a precursor for carbonaceous residue formation. Furthermore, the presence of casein-rich regions throughout the composite structure may facilitate the development of a protective char layer during combustion, contributing to the reduction in heat release parameters.

### 3.2. Data from TG/DTG/DSC Analysis

**Figure 2:**
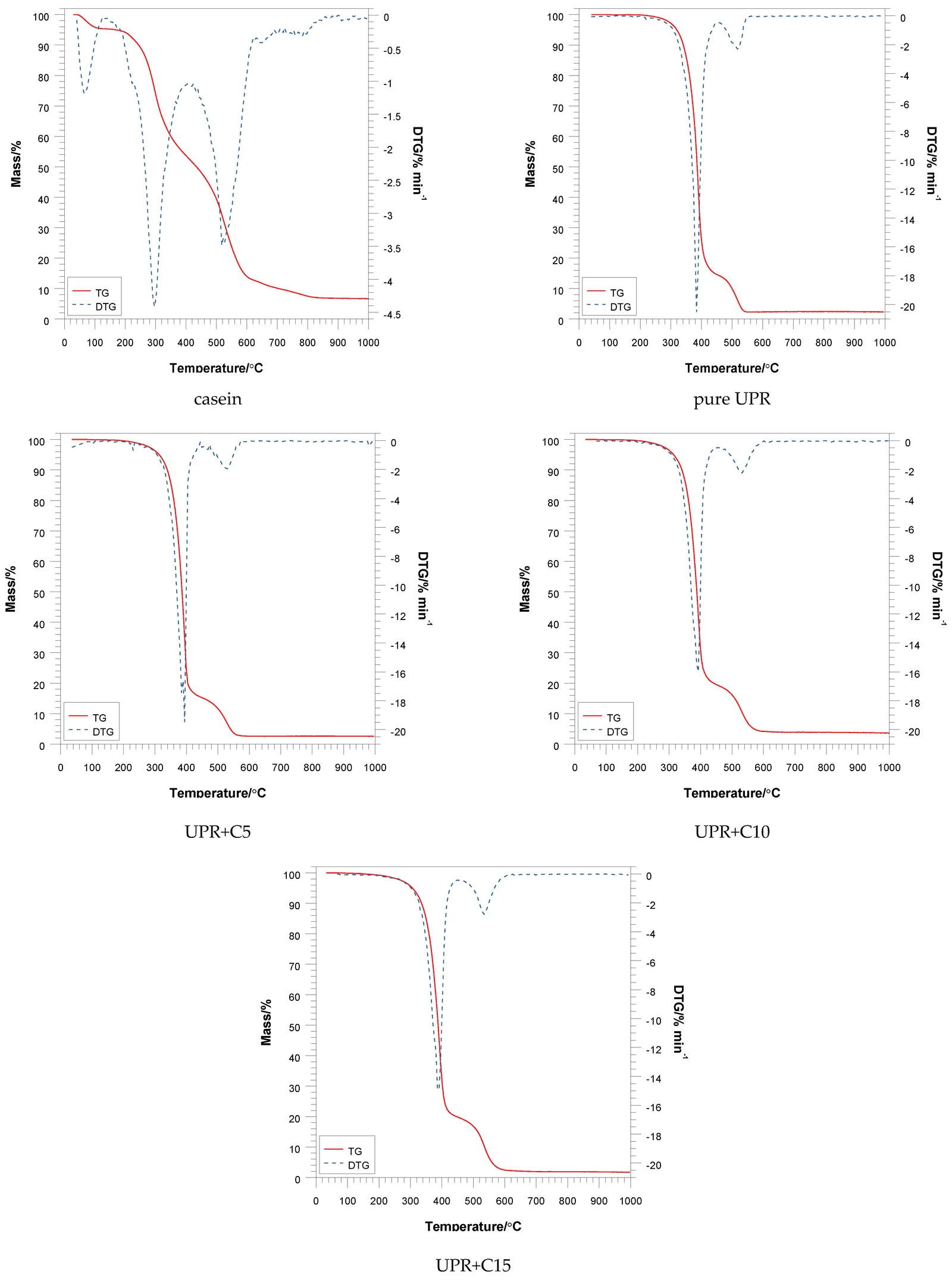
Thermal decomposition of casein powder, pure UPR, and casein-based biocomposites.

The thermal degradation behaviour of pure UPR, casein powder, and casein-modified composites was investigated using thermogravimetric (TG), derivative thermogravimetric (DTG), and differential scanning calorimetry (DSC) analyses. The obtained thermal parameters are summarized in Table 1, while the corresponding TG/DTG curves are presented in Figure 2. For samples exhibiting more than one thermal degradation stage, the values of T_max_, Δm, and T_p_ are presented in the same sequence, with each set of values corresponding to the same degradation stage or thermal event. Thus, the first T_max_, Δm, and T_p_ values refer to the first degradation stage, while subsequent values correspond to the following stages. This arrangement allows the thermal events identified from the DTG and DSC curves to be directly compared.

Pure UPR exhibited a typical two-stage mass-loss degradation process, which was also observed for all casein-containing composites. The first stage was associated primarily with the decomposition of the polyester matrix, whereas the second stage corresponded to further degradation of the remaining decomposition products and carbonaceous residue. Increasing the casein content resulted in a gradual increase in residual mass at 1000 °C, from 1.7% for UPR+C5 to 3.6% for UPR+C15, whereas practically no residue remained for pure UPR. The increased amount of carbon residues in the composites was probably due to a different degradation mechanism. Under oxidative conditions, this behaviour can be attributed to the formation of a protective layer on the sample surface during combustion, which acted as a protective char layer [24]. This layer reduced the evaporation and diffusion of combustible gases into air, and at the same time reduced heat release and mass loss rates, thereby enhancing the thermal stability of the composites during the later stages of degradation [25].

Previous studies have indicated that the type and concentration of fillers or modifying components can influence the thermal degradation behaviour and characteristic temperatures of polymer composites [26].

For pure unsaturated polyester resin, the temperatures corresponding to 5, 10, 50, and 80% mass change were 317, 341, 386, and 410 °C, respectively. The incorporation of casein resulted in a slight reduction in the initial degradation temperatures. The T5% and T10% values decreased gradually with increasing casein content, reaching 306 and 335 °C for the UPR+C15, respectively. This behaviour can be associated with the lower thermal stability of the protein-based casein components, which undergo decomposition at lower temperature range than the polymer matrix.

Despite the earlier onset of degradation, the composites exhibited improved resistance during the advanced stages of thermal decomposition. The temperature corresponding to 80% mass loss increased from 410 °C for pure UPR to 446 °C for UPR+C15. Similarly, the final decomposition temperature increased from 535 °C to 564 °C. These changes, together with the increase in residual mass, indicate that the incorporation of casein modifies the thermal decomposition behaviour of the UPR matrix and promotes the formation of decomposition products that are more resistant to further mass loss under the applied experimental conditions.

The DTG curves showed that the maximum degradation temperature of the first major decomposition stage remained relatively similar for the investigated materials, ranging from 384 °C for pure UPR to 385 °C for UPR+C15. In contrast, the second DTG peak shifted towards higher temperatures with increasing casein content, from 519 °C for pure UPR to 535 °C for UPR+C15. This shift indicates that the decomposition processes occurring at higher temperatures are affected by the presence of casein-derived products. The increase in the second DTG peak temperature, together with the higher residual mass, may be associated with the formation of thermally more resistant carbonaceous products during decomposition. Nevertheless, the present measurements do not provide sufficient evidence to identify the specific mechanism responsible for this behaviour.

Overall, the effect of casein on the thermal behaviour of UPR is characterized by a slight decrease in the temperatures associated with the initial mass loss and an increase in the characteristic temperatures and residual mass at later stages of decomposition. Thus, casein does not produce a uniform shift in thermal stability across the entire decomposition range. Instead, its incorporation changes the overall decomposition behaviour of the UPR matrix, particularly during the later stages of thermal degradation. Because the measurements were performed at a single heating rate, these observations should not be interpreted as evidence of changes in degradation kinetics or as a determination of kinetic parameters.

DSC analysis was additionally used to characterize the thermal events occurring in the investigated materials as a function of temperature. The fact that casein undergoes thermal degradation in air indicates a complex nature of the degradation mechanism with two degradation domains, namely endothermic and exothermic [27]. The thermal behaviour of casein powder showed several characteristic peaks. The first endothermic peak at 67 °C was attributed to the removal of physically absorbed water (3.6%). Additional endothermic peaks observed at 227 and 298 °C may be associated with changes and decomposition of protein structures, whereas the higher-temperature exothermic peaks can be related to further decomposition and carbonization processes (Table 1).

For cured pure UPR, a broad exothermic region was observed between approx. 300 and 550 °C, with two maxima at 386 °C and 503 °C. In the casein-containing composites, the corresponding high-temperature thermal events occurred at progressively higher temperatures. For the UPR+C15, the maxima were observed at 403 °C and 527 °C. These shifts are consistent with the TG/DTG results and indicate changes in the thermal decomposition behaviour of the composites at higher temperatures. In combination with the increased residual mass, the results suggest the formation of thermally more resistant decomposition products in the presence of casein.

### 3.3. HDT/VST of Biocomposites

The thermomechanical behaviour of the unsaturated polyester resin composites containing casein was evaluated by determining the Vicat softening temperature (VST) and heat deflection temperature (HDT). The obtained values are summarized in Table 2.

The Vicat softening temperature measurements were limited by the maximum operating temperature of the silicone oil bath in the Instron CEAST HV3 tester. For pure UPR, the specified penetration depth of 0.72 mm was not reached within the available measurement range; therefore, its actual VST could not be determined and is reported as >150 °C. The value of 150 °C represents only the upper instrumental measurement limit and was not treated as the actual VST of pure UPR.

For the casein-containing composites, the VST values were 142.95 ± 0.25 °C, 136.25 ± 0.25 °C, and 133.80 ± 0.10 °C for UPR+C5, UPR+C10, and UPR+C15, respectively. Thus, the incorporation of casein resulted in a progressive decrease in the temperature at which the specified penetration criterion was reached. Statistical analysis confirmed significant differences among the casein-containing formulations (one-way ANOVA, *p* < 0.05; Tukey’s HSD test). Because the VST of pure UPR was above the instrumental measurement range, it was not included as a quantitative value in the statistical comparison.

In contrast, the HDT increased progressively with increasing casein content, from 56.85 °C for pure UPR to 57.91 °C, 70.85 °C, and 71.31 °C for UPR+C5, UPR+C10, and UPR+C15, respectively. This corresponds to increases of approximately 1.9%, 24.6%, and 25.4%, respectively. One-way ANOVA followed by Tukey’s HSD multiple-comparison test indicated statistically significant differences among all investigated formulations (*p* < 0.05).

The increase in HDT suggests that the incorporation of casein restricts thermally induced deformation of the UPR matrix, possibly through increased interfacial interactions and reduced segmental mobility of the polymer network. The different trends observed for VST and HDT can be attributed to the different mechanical principles underlying the two measurements. VST is based on localized penetration under a specified load, whereas HDT characterizes the temperature at which a specimen reaches a specified deformation under flexural loading [28]. However, the opposite trends in these parameters may also reflect differences in the local and bulk structural response of the casein-containing network. In our previous study on casein/UPR composites, the incorporation of casein increased the calculated crosslinking density from 1430.9 mol m^−3^ for pure UPR to 1976.8 and 2279.7 mol m^−3^ for composites containing 5 and 15 wt% casein, respectively. In parallel, the storage modulus in the rubbery plateau increased from 16.2 to 22.3 and 25.8 MPa, respectively [23]. These results indicate that casein incorporation promotes a more constrained cured network and limits polymer-chain mobility, which can contribute to the higher resistance to thermally induced deformation and consequently to the increase in HDT.

At the same time, the presence of casein particles introduces additional particle–matrix interfaces and structural heterogeneity. Although these features can enhance the overall stiffness and constrain the polymer network, they may also affect the local deformation response under the concentrated load applied during the VST measurement. Local differences in stiffness and interfacial characteristics may facilitate localized deformation or penetration before substantial bulk deformation occurs. This interpretation is consistent with the previously observed increase in flexural modulus from 3.66 to 3.85 GPa with increasing casein content, accompanied by a decrease in flexural strength from 76.58 to 61.28 MPa and impact strength from 8.059 to 2.751 kJ m^−2^ [23]. Thus, casein reinforcement increases the stiffness and structural constraint of the cured material while simultaneously reducing its ability to accommodate localized deformation. Consequently, the higher HDT and lower VST should not be considered contradictory, but rather as a consequence of the different deformation scales and loading conditions involved in the two measurements.

### 3.4. Data from Cone Calorimetry

Cone calorimetry is a widely used method for evaluating the fire behaviour of polymeric materials, providing crucial information about ignition, heat release, smoke generation, and combustion residue formation [29,30,31,32]. The obtained parameters for pure UPR and casein-modified composites are summarized in Table 3.

The incorporation of casein significantly influenced most of the investigated combustion parameters of UPR composites, resulting in a composition-dependent reduction in heat release parameters. One-way ANOVA revealed no statistically significant effect of casein content on the time to ignition (TTI) (F(3,8) = 2.00, *p* = 0.193). The TTI decreased slightly from 32 s for pure UPR to 29 s for UPR+C15. Thus, although a gradual decrease in TTI was observed with increasing casein content, this change was not statistically significant. This behaviour is consistent with thermogravimetric (TG) results, where a slight reduction in the initial degradation temperature was observed due to the lower thermal stability of protein-based components.

Despite the minor decrease in TTI, the addition of casein considerably reduced combustion intensity. The peak heat release rate (pHRR) decreased progressively from 600 kW m^−2^ for pure UPR to 499, 465, and 407 kW m^−2^ for UPR+C5, UPR+C10, and UPR+C15, respectively. One-way ANOVA confirmed a highly significant effect of formulation on pHRR (F(3,8) = 86.06, *p* < 0.001). Tukey’s HSD test showed that pure UPR differed significantly from UPR+C5, UPR+C10, and UPR+C15, while UPR+C5 and UPR+C10 did not differ significantly from each other. The UPR+C15 composite exhibited a significantly lower pHRR than all other formulations. Overall, the pHRR was reduced by approximately 32% for UPR+C15 compared with pure UPR.

A similar statistically significant trend was observed for the total heat release (THR), which decreased from 86 MJ m^−2^ for pure UPR to 79, 71, and 65 MJ m^−2^ for UPR+C5, UPR+C10, and UPR+C15, respectively. The effect of formulation on THR was highly significant (F(3,8) = 77.77, *p* < 0.001), and Tukey’s multiple-comparison analysis indicated significant differences between all formulations. Thus, increasing the casein content resulted in a progressive and statistically significant reduction in the total amount of heat released during combustion.

The maximum average rate of heat emission (MARHE) was also significantly affected by casein incorporation (F(3,8) = 31.02, *p* < 0.001). MARHE decreased from 345 kW m^−2^ for pure UPR to 325, 283, and 255 kW m^−2^ for UPR+C5, UPR+C10, and UPR+C15, respectively. According to Tukey’s HSD test, pure UPR and UPR+C5 did not differ significantly, whereas UPR+C10 and UPR+C15 showed significantly lower MARHE values. Furthermore, the difference between UPR+C10 and UPR+C15 was statistically significant. These results indicate that increasing the casein content, particularly at 10 and 15 wt%, effectively reduced the intensity of heat release during combustion.

The reduction in heat-release parameters was accompanied by a significant increase in the amount of residue formed after combustion. Residue content increased from 0.1% for pure UPR to 1.4%, 2.6%, and 3.5% for UPR+C5, UPR+C10, and UPR+C15, respectively. The overall effect of formulation was statistically significant (F(3,8) = 11.58, *p* = 0.0028). Tukey’s test indicated that UPR+C10 and UPR+C15 produced significantly more residue than pure UPR, while the UPR+C15 formulation also showed a significantly higher residue content than UPR+C5. The progressive increase in residue formation suggests that casein promotes the formation of a more stable condensed-phase residue during thermal decomposition, which may contribute to the observed suppression of heat release.

In contrast to the heat-release parameters, the total smoke production (TSP) increased with casein incorporation, from 30.0 m^2^ for pure UPR to 33.5, 34.3, and 35.7 m^2^ for UPR+C5, UPR+C10, and UPR+C15, respectively. The overall effect was statistically significant (F(3,8) = 7.83, *p* = 0.009). Tukey’s HSD analysis showed that all casein-containing composites differed significantly from pure UPR, whereas no significant differences were observed among UPR+C5, UPR+C10, and UPR+C15. Therefore, the incorporation of casein was associated with a statistically significant increase in TSP compared with pure UPR; however, increasing the casein content above 5 wt% did not result in a further statistically significant increase in smoke production. The increased TSP may be related to changes in the decomposition pathway and volatile-product evolution associated with the protein-containing additive; however, further chemical analysis would be required to confirm the underlying mechanism.

These results indicate that the effect of casein on fire behaviour involves both beneficial and adverse aspects. On the one hand, casein substantially reduced pHRR, THR, and MARHE, indicating a lower intensity and total amount of heat release during combustion. On the other hand, these improvements were accompanied by increased smoke production relative to pure UPR. Therefore, the effect of casein should be considered in a broader fire-safety context, taking into account both heat release and smoke generation.

To further quantify the changes in fire performance, the Fire Performance Index (FPI) was calculated. The FPI increased from 0.053 m^2^·s kW^−1^ for pure UPR to 0.062, 0.065, and 0.071 m^2^·s kW^−1^ for UPR+C5, UPR+C10, and UPR+C15, respectively. One-way ANOVA demonstrated a significant effect of formulation on FPI (F(3,8) = 9.25, *p* = 0.0056). Tukey’s test showed that the FPI of UPR+C15 was significantly higher than that of pure UPR, whereas the UPR+C5 formulation did not differ significantly from pure UPR. The UPR+C10 formulation showed an intermediate response and was not significantly different from either UPR+C5 or UPR+C15. The progressive increase in FPI is primarily associated with the substantial reduction in pHRR, which more than compensates for the small, statistically non-significant decrease in TTI. These results indicate that casein modification improves the heat-release-related fire performance of the UPR matrix, mainly by reducing the intensity of heat release rather than by delaying ignition.

The effectiveness of the flame-retardant action was further evaluated using the Flame Retardancy Index (FRI). FRI increased from 1.00 for pure UPR to 1.27, 1.47, and 1.77 for UPR+C5, UPR+C10, and UPR+C15, respectively. According to the classification reported in the literature [5,31], these values correspond to an increasing flame-retardant effect. However, FRI is a calculated comparative index rather than a directly measured fire-safety parameter and should therefore be interpreted together with the individual cone calorimetry parameters.

The effect of casein content on FRI was statistically significant (F(3,8) = 16.65, *p* < 0.001). Tukey’s multiple-comparison test showed that UPR+C10 and UPR+C15 had significantly higher FRI values than pure UPR, while UPR+C15 also exhibited a significantly higher FRI than UPR+C5 and UPR+C10. The UPR+C5 formulation showed an intermediate value and did not differ significantly from either pure UPR or UPR+C10. The increase in FRI is consistent with the simultaneous reductions in pHRR and THR and the increase in residue formation.

Nevertheless, the FRI values should not be interpreted as evidence of an overall improvement in fire safety. The reduction in heat-release parameters was accompanied by a small, statistically non-significant decrease in TTI and a statistically significant increase in TSP. Thus, the results indicate that increasing the casein content primarily improves the heat-release-related fire behaviour of the UPR composites, while its effect on smoke generation represents an adverse aspect that should be considered in a comprehensive fire-safety assessment. In particular, the FRI value of 1.77 obtained for UPR+C15 should be interpreted as an indicator of improved flame-retardant performance relative to pure UPR under the investigated test conditions, rather than as evidence of “good” fire safety at the application level.

Overall, the statistical analysis demonstrates that casein incorporation primarily affects the combustion stage following ignition. While the change in TTI was not statistically significant, substantial and statistically significant reductions in pHRR, THR, and MARHE were observed, accompanied by increases in FPI, FRI, and residue formation. These beneficial changes were, however, associated with increased total smoke production relative to pure UPR. Among the investigated formulations, UPR+C15 exhibited the most pronounced reduction in pHRR, THR, and MARHE and the highest FPI, FRI, and residue content, while its TSP was not statistically different from those of UPR+C5 and UPR+C10. Thus, UPR+C15 provided the most pronounced improvement in heat-release-related fire performance among the investigated formulations, although the associated increase in smoke production should also be considered when evaluating its overall fire-safety performance. These results suggest that the enhanced heat-release characteristics are mainly associated with the formation of a protective condensed-phase residue and the consequent limitation of heat release during combustion.

**Figure 3:**
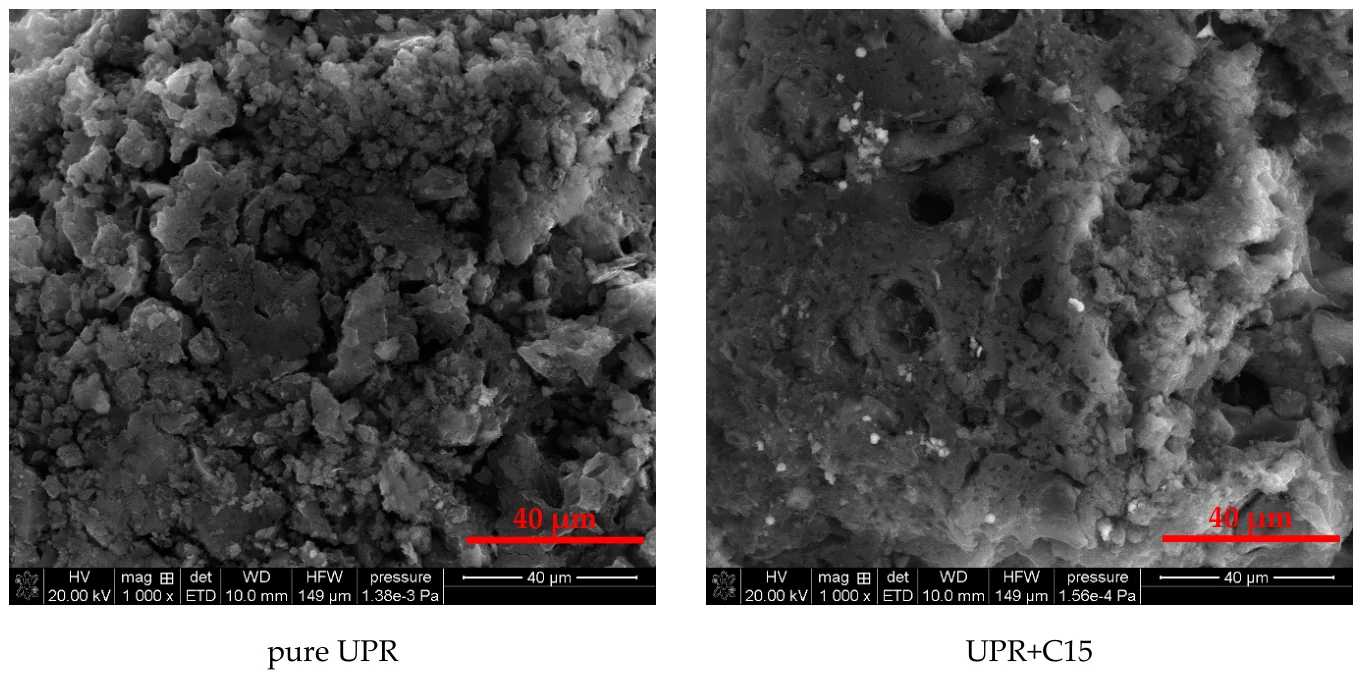
SEM micrographs showing the morphology of char residues obtained after cone calorimetry test of pure UPR and the UPR+C15 composite (magnification 1000×).

Scanning Electron Microscopy (SEM) observations of the combustion residues (Figure 3) provided direct visual confirmation of these quantitative findings, revealing clear morphological differences between the samples. The char residue obtained from pure UPR exhibited a loose, highly fractured, and heterogeneous structure with numerous voids and discontinuities. Such a brittle, non-continuous char layer provides limited physical protection, allowing relatively unhindered heat transfer and fuel diffusion into the flame zone.

In contrast, the residue from UPR+C15 composite showed a far more compact, continuous, and integrated carbonaceous matrix. While the surface features distinct macropores resulting from the release of non-flammable gases during swelling, the underlying matrix remains intact. Additionally, the micro-sized bright inclusions uniformly distributed across the UPR+C15 char surface correspond to the mineralized or phosphorylated residues of the decomposing protein. This cohesive morphology supports the cone calorimetry results and suggests that casein promotes the formation of a stable, dense protective barrier.

Considering both the thermal analysis and fire behaviour results, it can be concluded that the addition of casein to the resin facilitates the formation of a thermally stable char layer up to 600 °C [23]. As casein decomposes under the influence of heat, it triggers an early charring process that may contribute to limiting the thermal exposure of the underlying polymeric matrix. Furthermore, the pyrolysis reaction produces greater amounts of both non-flammable by-products and protective carbonaceous char.

This surface char layer acts as a physical barrier, reducing heat and mass transfer between the condensed and gas phases, a phenomenon known as the condensed-phase mechanism [10]. The presence of nitrogen-containing protein structures and phosphorus-containing groups within the casein chemistry may synergistically facilitate this behaviour. Overall, the results demonstrate that casein is a promising bio-based additive for reducing heat release in unsaturated polyester resin composites. However, its potential benefits should be considered together with its effect on smoke generation, which represents an important aspect of fire safety [33].

### 3.5. Flammability of Biocomposites

The combustion behaviour of polymeric materials depends on a complex interaction between heat, oxygen, and the generation of the combustible degradation products. During heating, polymer decomposition generates volatile compounds, condensed products, and carbonaceous residues. Flame propagation can be effectively restricted by impeding heat transfer to the condensed phase, decreasing the emission rate of combustible gases, or promoting the formation of an uninterrupted protective char barrier.

#### 3.5.1. Horizontal Flammability

The quantitative parameters derived from the horizontal flammability tests are summarized in Table 4. All investigated specimens completely across the entire measuring length without exhibiting melt dripping or detachment of flaming fragments. Regardless of the casein content, all composites fell under the HB40 classification, which correspond to a linear burning rate strictly below 40 mm·min^−1^ [34].

The pure UPR sample exhibited a linear burning rate of 23.44 mm·min^−1^. The incorporation of 5 wt.% casein raised the burning rate to 28.57 mm·min^−1^, representing a notable increase of approximately 22%. Further increasing in biopolymer loading led to a gradual reduction in the burning rate, yielding 26.55 and 26.32 mm·min^−1^ for UPR+C10 and UPR+C15, respectively. Although these values demonstrate a concentration-dependent improvement among the composites, they remained slightly elevated compared to pure UPR. Consequently, in contrast to the forced-flaming conditions in cone calorimetry, the addition of casein did not upgrade the horizontal flammability rating, as all formulations sustained the same HB40 classification under self-sustained horizontal burning conditions.

**Figure 4:**
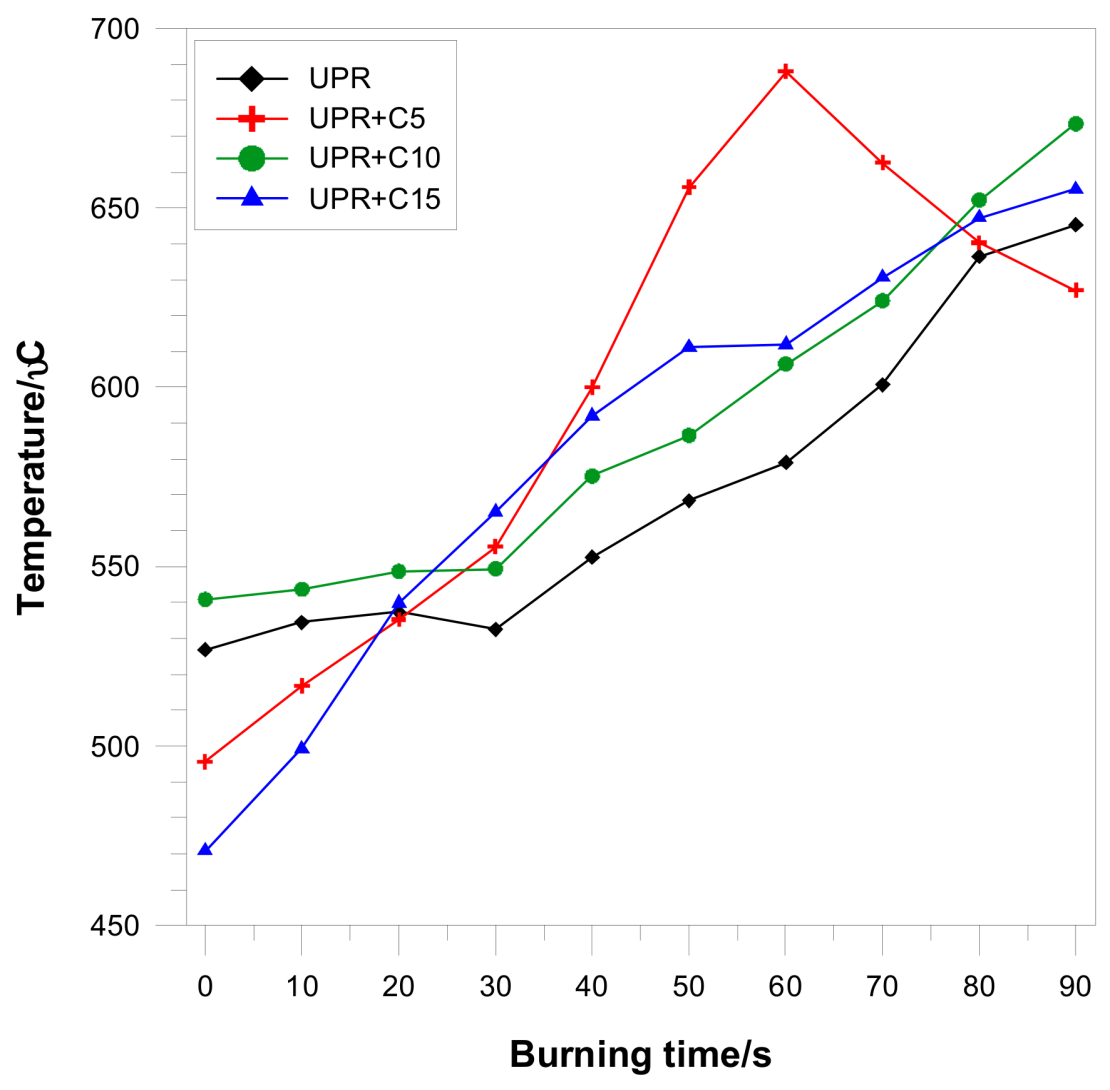
Maximum temperature of samples versus burn time.

To gain deeper insights into the combustion dynamics, thermal imaging analysis was conducted to monitor the maximum temperature profiles within the burning zone over time (Figure 4). The maximum temperature in the burning zone generally increased after ignition source removal, reflecting continued combustion and heat accumulation within the flame-affected region.

The highest temperature immediately after flame removal was recorded for the UPR+C10 composite, exceeding the value obtained for pure UPR by approximately 14 °C. In contrast, the UPR+C15 sample exhibited the lowest temperature in the burning region, approximately 11% lower than pure UPR. This behaviour suggests that higher casein loading contributes to a reduction in heat accumulation during combustion, which is consistent with the reduced pHRR and MARHE values obtained from cone calorimetry.

The analysis of thermal images showed that increasing combustion time resulted in the expansion of the heated area and changes in temperature distribution along the specimen surface. Supplementary thermal images (Figures S1–S4) and videos (Videos S1–S4) illustrating the combustion behaviour of pure UPR and casein-modified composites are provided.

#### 3.5.2. Vertical Flammability

**Figure 5:**
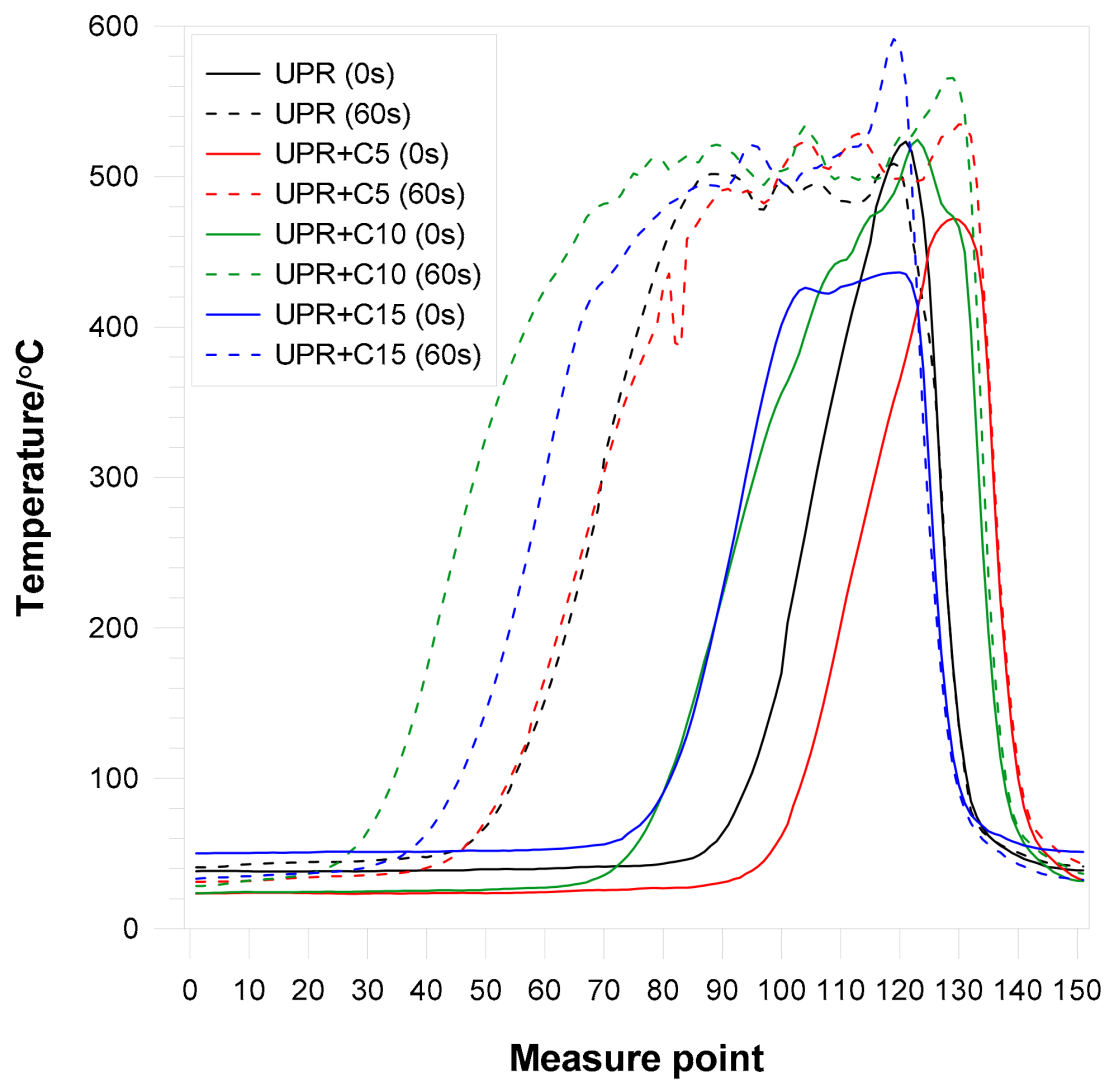
Temperature of samples in the vertical flammability test, immediately after the flame removal (time = 0 s) and after 60 s.

The temperature distribution profiles quantified along the longitudinal axis of the specimens during the vertical flammability test are detailed in Figure 5. The thermographic profiles illustrate the spatial propagation of the thermal wave immediately after the ignition source removal (0 s, solid lines) and after a subsequent burning period of 60 s (dashed lines). This spatial–temporal analysis tracks the progressive growth, shifting, and structural behaviour of the localized burning zone.

After 60 s of vertical flame propagation, the largest flame-affected areas were observed for UPR+C10 and UPR+C15 composites, whereas pure UPR and UPR+C5 exhibited smaller heated regions. These differences indicate that casein content influences heat distribution during combustion; however, the effect depends on the test configuration and burning conditions.

### 3.6. Proposed Flame-Retardant Mechanism of Casein-Modified UPR Biocomposites

Based on the comprehensive thermal analysis, forced-flaming cone calorimetry measurements, flammability tests, and morphological microstructural characterization of the char, a proposed flame-retardant mechanism for casein within the recycled PET-derived UPR matrix can be advanced. The experimental results suggest that the improved fire performance is primarily associated with the development of a more stable and cohesive condensed-phase residue, while possible complementary gas-phase effects may also contribute. However, the specific chemical reactions and gaseous intermediates involved in these processes were not directly characterized in the present study and therefore remain mechanistic hypotheses.

#### 3.6.1. Condensed-Phase Protective Action

The thermogravimetric analysis (TGA) results indicate that the incorporation of casein affects the initial thermal decomposition behaviour of the matrix. The observed reduction in the onset degradation temperatures (T_5%_ and T_10%_) can be attributed to the comparatively lower thermal stability of the protein-based components, which undergo primary decomposition at lower temperatures than the crosslinked polyester network.

Importantly, the thermal behaviour suggests a two-stage effect of casein: although casein decreases the initial decomposition temperatures (T_5%_ and T_10%_), it appears to promote greater thermal stabilization and residue formation at advanced stages of degradation. This is evidenced by the increase in T80% from 410 to 446 °C and the increase in residual mass from 0.1% for pure UPR to 3.5% for UPR+C15. Thus, the flame-retardant effect of casein does not appear to arise from suppression of the initial degradation process; rather, the results suggest a shift in the subsequent degradation pathway toward increased carbonaceous residue formation and development of a protective condensed-phase structure.

At advanced stages of thermal conversion, a possible interaction between the decomposing casein and the recycled PET-derived UPR matrix may further contribute to this behaviour. This matrix contains a substantial fraction of rigid aromatic phenyl rings originating from terephthalate segments, while casein contains reactive functional groups including amine, carboxyl, hydroxyl and amide functionalities. Under thermal stress, these functionalities may participate in dehydration, condensation, recombination, and cyclization reactions involving the surrounding polymer structure. Such interactions could facilitate the development of a more thermally stable, carbon-rich residue; however, the occurrence of specific aromatic co-crosslinking reactions was not directly verified in the present work and should therefore be regarded as a proposed mechanism.

The observed changes in T_80%_ (from 410 °C to 446 °C), the extension of the final decomposition temperature (T_f_ to 564 °C), and the gradual increase in residual mass from 0.1% for pure UPR to 3.5% for UPR+C15 are consistent with this interpretation. Nevertheless, these thermal parameters alone cannot establish the formation of specific chemical bonds or crosslinked structures.

The macroscopic efficacy of this dense, aromatically reinforced barrier is explicitly verified by the SEM microstructural observations (Figure 3). Whereas pure UPR yields a relatively brittle and discontinuous char characterized by deep cracks and macrostructural voids, the UPR+C15 composite promotes a more continuous, cohesive, and dense carbonaceous architecture. This morphology is expected to reduce the transport of volatile combustible degradation products from the underlying polymer to the flame zone and may also limit heat transfer toward the un-decomposed material. The improved barrier morphology is consistent with the reductions observed in the forced-flaming parameters, including approximately 32% reduction in pHRR, 24.4% reduction in THR, and a decrease in MARHE from 345 to 255 kW m^−2^. The progressive increase in the calculated FPI and FRI values further supports the beneficial effect of the modified char structure on fire-development behaviour.

#### 3.6.2. Proposed Gas-Phase Flame Inhibition and Radical Scavenging

In addition to the dominant condensed-phase contribution, a possible complementary gas-phase mechanism may arise from the chemical composition of casein. Casein is a phosphoprotein containing organically bound phosphorus together with nitrogen-rich protein structures. During thermal decomposition, phosphorus-containing groups may generate phosphorus-containing acidic or volatile species that can potentially participate in both condensed- and gas-phase processes.

Previous studies have proposed that phosphorus-containing species released during the thermal decomposition of phosphorous-containing polymers can generate reactive intermediates such as ${\mathrm{HPO}}_{2}^{*}$, HPO^*^, ${\mathrm{PO}}_{2}^{*}$, and PO^*^, which may interact with flame-propagating radicals such as H^*^ and OH^*^ [35,36]. Accordingly, a similar phosphorus-mediated radical-quenching pathway may be proposed for the present casein/UPR system.

Concurrently, the nitrogen-containing fragments present in casein may also contribute to flame suppression through the formation of volatile nitrogen-containing products during thermal decomposition. Such products, potentially including NH_3_ and other nitrogen-containing species, may contribute to dilution of the combustible degradation products and/or modification of the local flame environment.

The increase in total smoke production (TSP) observed during cone calorimetry may be consistent with changes in the combustion and degradation pathways; however, TSP alone cannot be used to confirm gas-phase radical scavenging or fuel-dilution mechanisms. Therefore, the gas-phase contribution is best regarded as a complementary hypothesis that may operate alongside the experimentally better-supported condensed-phase effect.

Overall, the fire-retardant behaviour of the casein-modified UPR composites can be proposed to arise predominantly from a condensed-phase protective mechanism, characterized by enhanced formation of a continuous and cohesive carbonaceous residue. The thermal and morphological results support the formation of this protective barrier, which is consistent with the observed reductions in pHRR, THR, and MARHE. In parallel, possible secondary gas-phase contributions from phosphorus- and nitrogen-containing decomposition products may further influence flame behaviour, although these pathways require direct chemical characterization for confirmation. Thus, the present results support a condensed-phase-dominated mechanism with potential complementary gas-phase effects, rather than establishing specific chemical reaction pathways.

## 4. Conclusions

The present study demonstrates that casein can be successfully incorporated as a sustainable bio-filler into an unsaturated polyester resin (UPR) matrix derived from recycled post-consumer poly(ethylene terephthalate) (rPET), providing simultaneous benefits in thermal, thermomechanical, and fire performance.

The main conclusions are as follows:

1. Casein particles were successfully incorporated and uniformly dispersed within the crosslinked UPR network, confirming the feasibility of producing homogeneous bio-based and circular UPR/casein thermosets.
2. Casein modified the thermal degradation behaviour of the composites. Although the initial decomposition temperature decreased slightly, the residual mass increased markedly, from 0.1% for pure UPR to 3.6% for UPR+C15, indicating enhanced char formation associated with the aromatic rPET-derived structure and protein fraction.
3. The thermomechanical response was method-dependent. At 15 wt.% casein, the HDT increased by 25.4%, from 56.85 °C to 71.31 °C, whereas the VST decreased from 150.0 °C to 133.8 °C.
4. Casein substantially improved fire performance under forced-flaming conditions. For UPR+C15, the pHRR and MARHE decreased by 32.2% and 26.1%, respectively, compared with pure UPR, accompanied by increased residue formation. These results indicate the development of a protective char layer that limits heat and mass transfer.
5. The fire performance indices confirmed the beneficial effect of casein. The FPI increased from 0.053 to 0.071 m^2^·s·kW^−1^, while the FRI reached 1.77 for UPR+C15, corresponding to good flame-retardant performance according to the adopted criteria.
6. Casein did not substantially alter the horizontal flammability classification, with all materials retaining HB40. The observed changes in burning behaviour and thermographic profiles further demonstrated the influence of sample orientation and heat-transfer mechanisms.
7. The enhanced fire performance is primarily attributed to a condensed-phase mechanism involving increased char formation and stabilization of a protective carbonaceous barrier, with additional contributions from gas-phase fuel dilution and radical trapping by phosphorus-containing species.

Overall, UPR+C15 exhibited the most favourable balance of thermal stability, thermomechanical performance, and flame-retardant properties. These findings demonstrate the potential of casein as a sustainable bio-based additive for improving the performance and fire safety of circular thermoset composites based on recycled PET.

Further research should focus on optimizing the UPR/casein formulation, improving interfacial compatibility, elucidating the mechanisms of char formation and flame retardancy, and assessing long-term durability, moisture resistance, mechanical performance, life-cycle impacts, and scalability.

## Figures and Tables

**Table 1:** TG/DTG/DSC data of pure UPR and casein-modified composites.

Sample	T_5%_ ^1^	T_10%_ ^2^	T_50%_ ^3^	T_80%_ ^4^	T_f_ ^5^	T_max_ ^6^	Δm ^7^	R ^8^	T_p_ ^9^
casein	169.1	243.9	434.1	563.7	813.9	65.3	−3.6	6.6	67.1
						223.1	−3.7		226.7
						297.2	−38.8		298.4
						528.7	−40.3		516.7
						783.7	−5.8		771.3
pure UPR	317.3	340.6	386.3	410.0	535.3	383.9	−85.0	—	385.8
						519.4	−14.0		502.7
UPR+C5	314.1	340.4	386.2	404.0	551.5	392.4	−82.6	1.7	400.6
						528.9	−14.7		512.3
UPR+C10	313.3	339.2	387.2	442.1	559.2	388.1	−80.3	2.5	401.5
						531.1	−15.6		518.0
UPR+C15	306.4	335.2	388.1	446.1	563.6	385.4	−77.8	3.6	403.1
						534.6	−16.9		526.5

^1^ Temperature at 5% mass loss, °C; ^2^ temperature at 10% mass loss, °C; ^3^ temperature at 50% mass loss, °C; ^4^ temperature at 80% mass loss, °C; ^5^ final temperature of decomposition, °C; ^6^ maximum decomposition temperature from DTG, °C (for samples exhibiting multiple degradation stages, the values are listed in the order of the corresponding degradation stages); ^7^ mass change associated with the corresponding degradation stage, %; ^8^ residual mass at 1000 °C, %; ^9^ DSC peak temperature corresponding to the respective thermal event, °C.

**Table 2:** Effect of casein content on the Vicat softening temperature (VST) and heat deflection temperature (HDT) of UPR composites.

Parameter	Sample			
	Pure UPR	UPR+C5	UPR+C10	UPR+C15
VST, °C	>150.00 *	142.95 ± 0.25 ^a^	136.25 ± 0.25 ^b^	133.80 ± 0.10 ^c^
HDT, °C	56.85 ± 0.05 ^a^	57.91 ± 0.10 ^b^	70.85 ± 0.15 ^c^	71.31 ± 0.10 ^d^

* The VST measurement for pure UPR was limited by the maximum temperature of the silicone oil bath (0.72 mm needle penetration depth at 150 °C); therefore, the actual VST may exceed the reported measurement range. Different superscript letters within the same row indicate statistically significant differences according to one-way ANOVA followed by Tukey’s HSD multiple-comparison test (*p* < 0.05).

**Table 3:** Cone calorimetry parameters, fire performance indices, and residue content of pure UPR and casein-modified UPR composites (mean ± SD, n = 3).

Sample	TTI ^1^	pHRR ^2^	THR ^3^	TSP ^4^	MARHE ^5^	FPI ^6^	FRI ^7^	Res ^8^
pure UPR	32 ± 2 ^a^	600 ± 12 ^a^	86 ± 2 ^a^	30.0 ± 1.5 ^a^	345 ± 15 ^a^	0.053 ± 0.004 ^a^	1.00 ^a^	0.1 ^a^
UPR+C5	31 ± 1 ^a^	499 ± 16 ^b^	79 ± 2 ^b^	33.5 ± 1.4 ^b^	325 ± 10 ^a^	0.062 ± 0.004 ^ab^	1.27 ± 0.12 ^ab^	1.4 ± 0.8 ^ab^
UPR+C10	30 ± 2 ^a^	465 ± 17 ^b^	71 ± 1 ^c^	34.3 ± 1.5 ^b^	283 ± 12 ^b^	0.065 ± 0.005 ^bc^	1.47 ± 0.16 ^bc^	2.6 ± 0.9 ^bc^
UPR+C15	29 ± 1 ^a^	407 ± 15 ^c^	65 ± 2 ^d^	35.7 ± 1.6 ^b^	255 ± 13 ^b^	0.071 ± 0.004 ^c^	1.77 ± 0.19 ^c^	3.5 ± 0.9 ^c^

^1^ Time To Ignition, s; ^2^ Peak Heat Release Rate, kW m^−2^; ^3^ Total Heat Release, MJ m^−2^; ^4^ Total Smoke Production, m^2^; ^5^ Maximum Average Rate of Heat Emission, kW m^−2^; ^6^ Fire Potential Index, m^2^ s kW^−1^; ^7^ Flame Retardancy Index; ^8^ Residue, %. Different superscript letters within the same column indicate statistically significant differences among formulations according to one-way ANOVA followed by Tukey’s HSD multiple-comparison test (*p* < 0.05). Values sharing at least one common superscript letter are not significantly different.

**Table 4:** Results of horizontal flammability tests of pure UPR and casein-modified UPR composites.

Sample	Burn Time of Measuring Length, s	Linear Burning Rate, mm min^−1^	Horizontal Flammability Classification
pure UPR	128	23.44	HB40
UPR+C5	105	28.57	HB40
UPR+C10	113	26.55	HB40
UPR+C15	114	26.32	HB40

## Data Availability

The dataset is available on request from the authors.

## Supplementary Materials

The following supporting information can be downloaded at https://www.mdpi.com/article/10.3390/polym18202552/s1, Figure S1: Thermal images of an unmodified sample (pure UPR); Figure S2: Thermal images of UPR+C5; Figure S3: Thermal images of UPR+C10; Figure S4: Thermal images of UPR+C15; Video S1: Combustion behaviour of pure UPR; Video S2: Combustion behaviour of UPR+C5; Video S3: Combustion behaviour of UPR+C10; Video S4: Combustion behaviour of UPR+C15.
