# Supplementary material for: Thermal, Thermomechanical, and Fire Behaviour of Casein-Modified Unsaturated Polyester Resin Biocomposites
Source: Polymers (Basel). 2026 Oct 16;18(20):2552. doi: 10.3390/polym18202552 (PMC13708879; doi:10.3390/polym18202552)
Supplement: Supplementary file 1 [file polymers-18-02552-s001.zip › Suppl Mater.pdf]

Thermal, Thermomechanical, and Fire Behaviour of Casein-Modified UPR Biocomposites

Przemysław Pączkowski [1,*], Karolina Głogowska [2], Bronisław Samujło [2], Barbara Gawdzik [1]

[1] Department of Polymer Chemistry, Institute of Chemical Sciences, Faculty of Chemistry, Maria Curie-Sklodowska University, Gliniana 33, 20-614 Lublin, Poland

[2] Department of Technology and Polymer Processing, Faculty of Mechanical Engineering, Lublin University of Technology, Nadbystrzycka 36, 20-618 Lublin, Poland

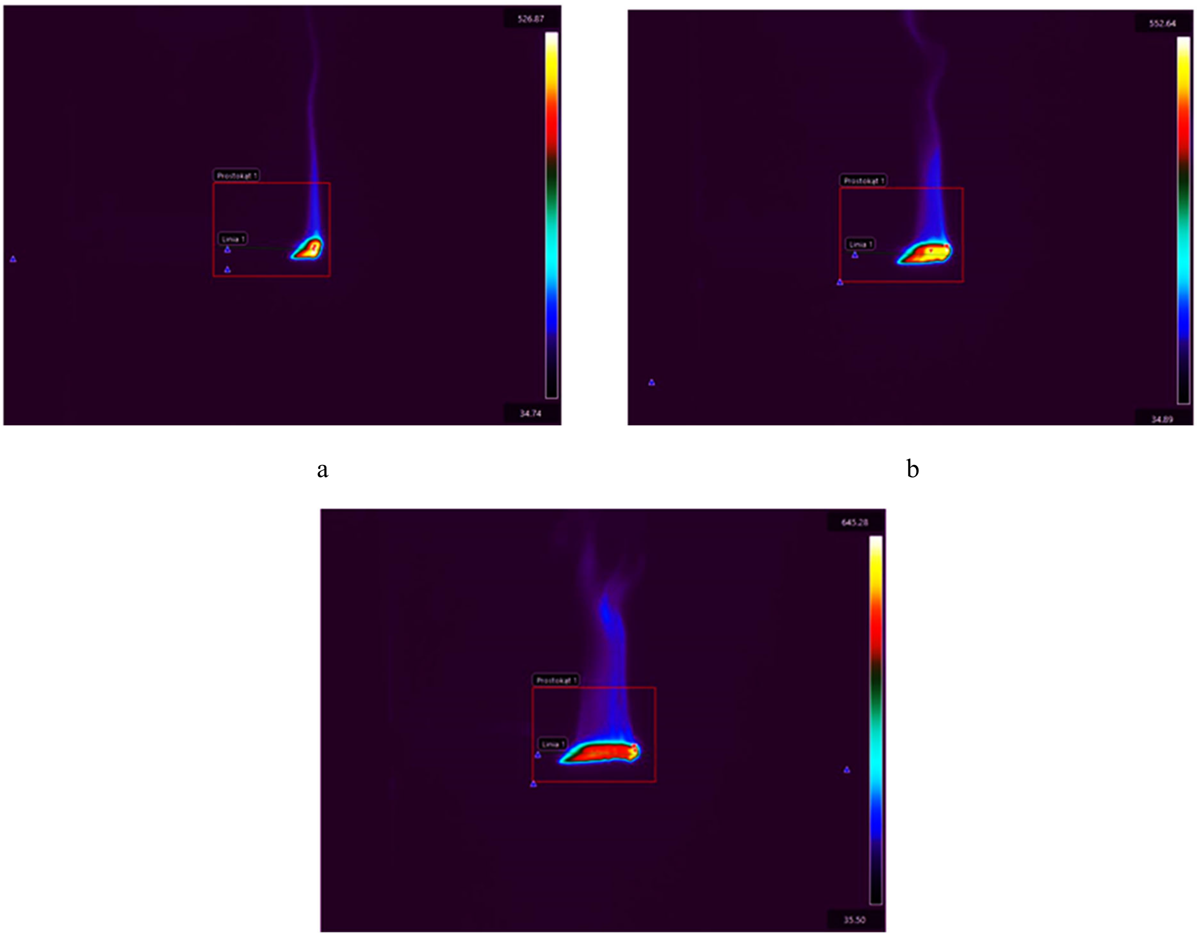

a b c

Figure S1. Thermal images of an unmodified sample (pure UPR): a) immediately after flame removal (time 0), b) after 40 seconds, c) after 90 seconds.

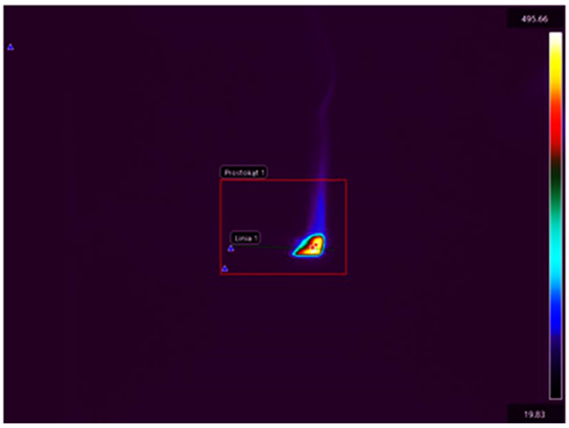

a

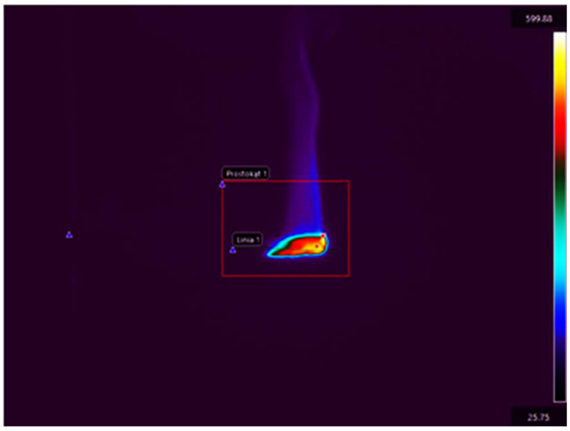

b

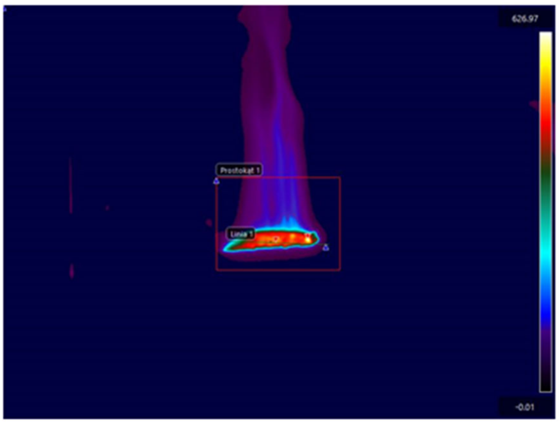

c

Figure S2. Thermal images of UPR+C5: a) immediately after flame removal (time 0), b) after 40 seconds, c) after 90 seconds.

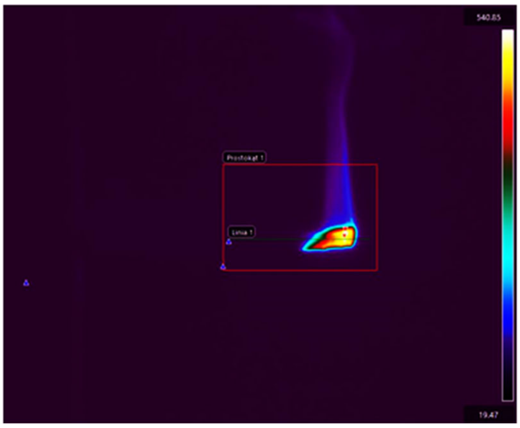

a

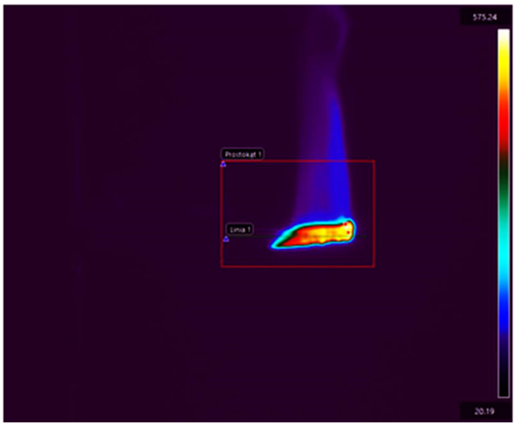

b

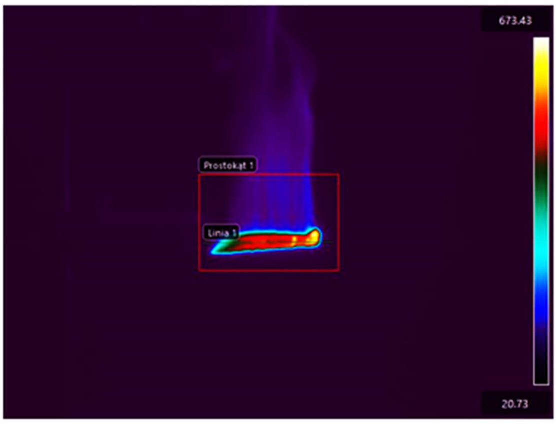

c

Figure S3. Thermal images of UPR+C10: a) immediately after flame removal (time 0), b) after 40 seconds, c) after 90 seconds.

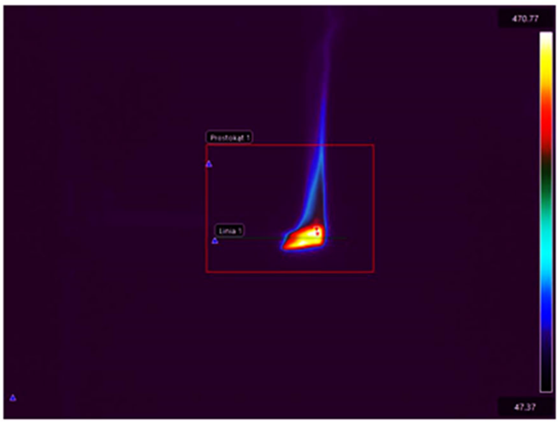

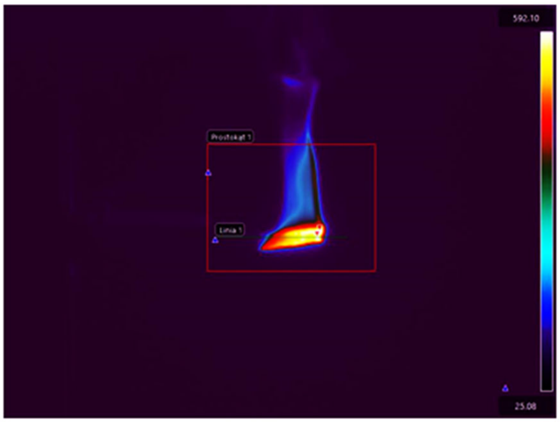

a b

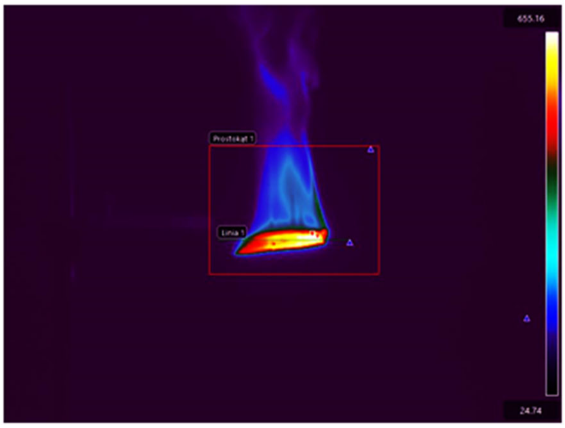

c

Figure S4. Thermal images of UPR+C15: a) immediately after flame removal (time 0), b) after 40 seconds, c) after 90 seconds.
